# Individual differences in social attachment: A multi‐disciplinary perspective

**DOI:** 10.1111/gbb.12792

**Published:** 2022-02-16

**Authors:** Morgan L. Gustison, Steven M. Phelps

**Affiliations:** ^1^ Department of Integrative Biology The University of Texas at Austin Austin Texas USA; ^2^ Institute for Neuroscience The University of Texas at Austin Austin Texas USA

**Keywords:** individual variation, levels of analysis, prairie vole, social attachment, social behavior, social neuroscience

## Abstract

Social behavior varies across both individuals and species. Research to explain this variation falls under the purview of multiple disciplines, each with its own theoretical and empirical traditions. Integration of these disciplinary traditions is key to developing a holistic perspective. Here, we review research on the biology of social attachment, a phenomena in which individuals develop strong affective connections to one another. We provide a historical overview of research on social attachment from psychological, ethological and neurobiological perspectives. As a case study, we describe work on pair‐bonding in prairie voles, a socially monogamous rodent. This specific topic takes advantage of many biological perspectives and techniques to explain social bonds. Lastly, we conclude with an overview of multi‐dimensional conceptual frameworks that can be used to explain social phenomena, and we propose a new framework for research on individual variation in attachment behavior. These conceptual frameworks originate from philosophy, physics, ethology, cognitive science and neuroscience. The application and synthesis of such frameworks offers a rich opportunity to advance understanding of social behavior and its mechanisms.

## Graphical abstract

We review research on the biology of social attachment, a phenomena in which individuals develop strong affective connections to one another. We provide a historical overview of research on social attachment from psychological, ethological and neurobiological perspectives, and we provide an overview of multi‐dimensional frameworks to explain social phenomena. The application and synthesis of such frameworks offers a rich opportunity to advance understanding of social behavior and its mechanisms.

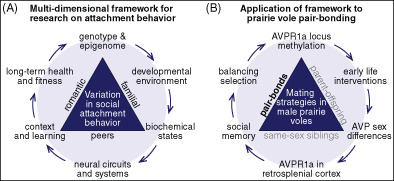

## 1 INTRODUCTION

The understanding of behavior is a daunting task. The execution of a behavior entails a series of causal steps that begin at the level of genomes and cells, through circuits and systems, to the dynamic modification of actions in response to a changing world. Among behaviors, perhaps none is more complex than social behavior, with its rapidly changing demands and its need for strategic updating based on the behaviors of conspecifics. Social behavior is also essential to many species, including our own. Given both its importance and its daunting complexity, it is perhaps not surprising that a variety of disciplines have focused on social behavior and its mechanisms, including psychology, anthropology and animal behavior, to name a few. The scientific understanding of social behavior requires synthesis of these disparate traditions into cogent explanatory frameworks.

This review uses the study of attachment behavior as a case study in the broader ambitions of social neuroscience. We briefly survey disciplinary traditions, levels of analysis, and prospects for future synthesis in both human and animal models. In doing so, we follow other authors who have emphasized the importance of multi‐scale analysis of behavior and its mechanisms.^1^ We add, however, that synthesis across traditions offers an unusually rich opportunity for insight and advance, as distinct disciplines bring different assumptions and interests to bear on related questions.

The study of social attachment and its mechanisms has a uniquely rich set of theoretical and empirical traditions. Perhaps at its simplest, one can focus on two traditions: that of psychology, whose primary interest is in understanding principles of human behavior^2^, ^3^; and that of ethologists, whose interests lie in understanding the natural diversity of behavior within and among animal species.^4^, ^5^ There are, of course, finer distinctions to be made—traditions in psychiatry, cognition or neuroscience, or anthropology and behavioral ecology, for example. Each of these areas has its own unique set of assumptions, interests and tools. Nevertheless, the principles that apply to one must surely apply to another, and so looking across disciplines holds tremendous promise.

**FIGURE 1:**
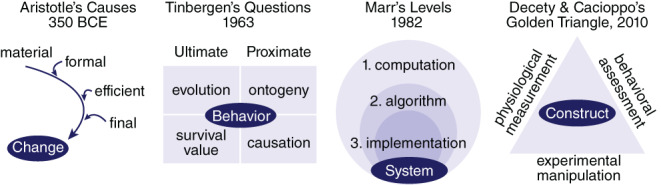
Multi‐dimensional conceptual frameworks used to explain physical, biological and cognitive phenomena

One interesting example of how much these disciplines have in common is how they have all struggled with the idea of what it means to “explain” a behavior (Figure 1). Their answers are parallel in many respects, and owe a debt, either explicit or implicit, to classic philosophy and the origins of Western science. Aristotle suggested there were four kinds of causes: material, formal, efficient, and final.^6^ The evolutionary biologist and pioneering ethologist Julian Huxley drew from Aristotle's insights to suggest three aspects of causation for animal behavior—mechanistic‐physiological, adaptive‐functional, and an evolutionary or historical aspect.^7^, ^8^ In his famous 1963 paper, Tinbergen reformulated Huxley's causation and added the ontogeny of behavior as a fourth causal force.^9^ This led to a tidy two‐by‐two framework for considering Tinbergen's four questions, in which behavior is described at the level of causation, survival value, ontogeny and evolution. Indeed, this framework has proved useful for interdisciplinary thinking. Like Huxley and Tinbergen, the computer scientist David Marr wondered what constitutes an explanation for behavior. He arrived at three levels: computation, algorithm, and implementation.^10^

Despite their obvious distinctions, these approaches have a variety of perspectives in common (Figure 1). Like Aristotle, they make distinctions between immediate or material causes—the exact ways in which matter is arranged and moved to produce a behavior—and the principles that guide how and why behaviors are executed. The material causes are of tremendous interest. As has been discussed elsewhere, the description of immediate causes is often taken as the most essential form of explanation in neuroscience.^1^ But our aspirations should extend beyond mechanisms. Principles guide theory and experiment, make novel predictions, and allow us to make inferences about novel situations or species.

The principles that are sought by each of these disciplines are not the same, but at some point they must converge. Cognitive neuroscience searches for algorithms, for example, but also recognizes the essential role of the body in shaping cognition.^11^ Psychologists look to animal models to understand principles of human thought and action. Behavioral ecologists examine neural and molecular mechanisms of behavior and their evolution. Each of these academic silos has its own unique insights, its own superstitions and suppositions, and yet the principles that underlie behaviors across species must have common threads. Humans are one example in a much larger set of outcomes.

Attachment is essential to human health and well‐being.^12^, ^13^ It is also widespread in the animal world—with bonds among parents and young, among mated pairs, among kin and nonkin—bonds that vary in strength based on the natural history of species and the unique demands of their lifestyles.^14^ They have been studied by social psychologists, by primatologists, ornithologists and many more. What are the common threads, if any? And what novel questions does a cross‐disciplinary, multi‐scale perspective lead us to ask? In the first part of this review, we synthesize research traditions on the sociobiological basis of attachment behavior, with an emphasis on individual variation. We follow this discussion with a case study on the integrative mechanisms that underlie pair‐bonding variation in prairie voles, a socially monogamous rodent. Then, we transition into a broader discussion of multi‐dimensional frameworks and how they can provide insights into social phenomena like attachment. We use these established frameworks, in conjunction with our research surveys, to inspire a new multi‐dimensional framework for exploring why individuals differ in attachment behavior.

## 2 A BRIEF HISTORY OF RESEARCH ON SOCIAL ATTACHMENT

**FIGURE 2:**
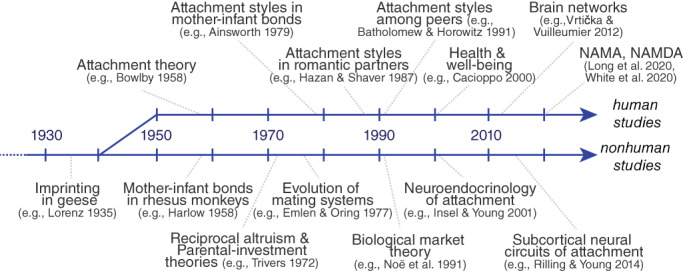
Timeline of research related to social attachment and behavior, featuring key discoveries, theories and research trends from the 1930s to present day. These accomplishments are split by traditions that emphasize human or nonhuman animal models

A fundamental component of the human experience, and the experience of many other species, is the capacity to form an enduring emotional connection, or “attachment,” with another individual. The question of why individuals form attachments has inspired scientific inquiry from a diverse array of disciplines for nearly a century (Figure 2). The empirical study of social attachment has its origins in the 1930s, when ethologist Konrad Lorenz made the seminal observation that goslings imprint on the first large moving object they encounter.^4^, ^5^ This finding was an early recognition that the drive to form attachments emerges in early life as a biologically pre‐programmed trait. Then, in the 1950s and 60s, separate lines of research from social psychologist John Bowlby and comparative psychologist Harry Harlow converged on the conclusion that early attachments, like the mother–infant bond, are essential components of healthy development.^15^, ^16^, ^17^, ^18^, ^19^, ^20^

In his “attachment theory,” John Bowlby makes the argument that human infants are predisposed to form attachments with a primary caregiver because such attachments are necessary for survival.^2^, ^3^ Bowlby's theory was derived from observations of child delinquents and hospitalized children, for which disruption of early life attachments appeared to negatively impact their behavioral and cognitive development.^21^ Then, Harry Harlow investigated the role of early life attachments by experimentally manipulating early life social exposure in rhesus macaques. In one line of work, Harlow found that mother–infant bonds functioned as more than just a way to provide nourishment. Infant monkeys preferred artificial doll “caregivers” covered with warm furry cloth to wire dolls that merely provided food.^18^, ^20^ In another line of work, Harlow found that early life social exposure is key to later behavioral and cognitive functioning. Monkeys who were chronically isolated became catatonic and incapable of interacting with others.^19^

Taken together, research by Lorenz, Bowlby and Harlow demonstrated that humans and other species have a biological predisposition to form social attachments, which in turn, sets the stage for healthy behavioral and cognitive development. A limitation of these studies, however, was that they treated social attachments as a binary variable, in which attachments either did or did not occur. This limitation fueled scientists to begin exploring social attachment as a continuous variable that varies across individuals (Figure 2). This research focus on individual variation was spearheaded in the 1960s by developmental psychologist Mary Ainsworth, who characterized attachment “styles” of mother–infant bonds.^22^ Ainsworth found that human infants show different patterns of exploratory and affective behaviors during a series of situations in which an infant's mother or a stranger was present in the same room with them.^23^ Based on these observations, Ainsworth theorized that infants express one of three main attachment styles (i.e., secure, avoidant and ambivalent), which could be attributed to variability in how attuned mothers were to their infant's needs.^24^, ^25^

With the foundation of attachment theory set by Lorenz, Bowlby, Harlow, Ainsworth and others, modern approaches continue to tackle a spectrum of questions about the causes and consequences of individual variation in social attachment. Attachment is a multi‐dimensional phenomena in that research questions can span a wide range of academic disciplines and levels of analysis. Generally, there has been little cross‐talk among these different modern approaches. Therefore, in this review, we seek to synthesize disjointed approaches in order to achieve an integrative understanding of why the capacity and motivation to form social bonds varies across individuals. We begin by surveying the general contributions made in human‐focused (e.g., social psychology) and nonhuman‐focused (e.g., ethology and comparative psychology) research at ultimate and proximate levels of analysis. Then, we discuss research seated at the interface of these disjoint approaches and potential opportunities for interdisciplinary work, specifically highlighting research on pair‐bonding in prairie voles (*Microtus ochrogaster*). Finally, we synthesize the current state of attachment research by structuring it onto philosophical frameworks used to explain multi‐dimensional phenomena. We use this holistic framework to identify future research directions.

## 3 VARIATION IN SOCIAL ATTACHMENT BEHAVIOR AMONG PEOPLE

One approach to studying attachment follows in the footsteps of Bowlby and Ainsworth in the academic disciplines of social and developmental psychology. In this approach, research has focused primarily on identifying how attachment relates to health outcomes at various developmental stages and social contexts (Figure 2). Below, we review how healthy social attachments are characterized and their impact on mental and physical wellbeing. Then, we summarize neuroscience research that has advanced understanding of the cognitive underpinnings of healthy attachment styles.

### 3.1 Functional role of attachment behavior in health and wellbeing

Attachment styles have been applied at several life history stages, from parent–child to adolescent and adult relationships.^25^, ^26^, ^27^, ^28^ Human attachment styles are broadly classified into two categories—secure and insecure—that are identified based on observed or reported patterns of interaction.^25^, ^29^ Generally, a secure attachment style is thought to encompass healthier and more stable ways of connecting to others, whereby individuals trust and are supported by their social partners.^24^, ^30^, ^31^ Behavioral patterns of secure attachment involve autonomy as well as high levels of proximity seeking, especially in times of distress. During the Strange Situation Procedure, for example, infants classified as having a secure attachment style readily leave their caregiver's side to explore the environment, but will approach the caregiver after being fully separated.^25^ Hazan and Shaver^28^ developed the Adult Attachment Interview to probe similar dimensions of adult relationships. As in infants, secure attachment in adult relationships implies that partners are comfortable with both intimacy and autonomy.^27^, ^28^, ^30^

Insecure styles of attachment, on the other hand, are used to classify more unstable patterns of interaction; these patterns involve individuals who are avoidant or anxious in their social attachments.^32^ During the Strange Situation Procedure, infants are classified into different insecure styles (i.e., avoidant and anxious‐ambivalent) based on their behavior during reunion with a caregiver.^24^, ^30^ A disorganized style applies to instances where infants or children show mixed behavioral patterns, which may occur when a caregiver causes both comfort and distress.^33^ Adults are categorized into similar styles (i.e., dismissive, preoccupied and unresolved‐disorganized) based on how they answer questions about their relationships.^27^, ^28^, ^29^, ^34^ Both secure and insecure styles of attachment are thought to reflect behavioral strategies that enable individuals to adapt to their social environment.^35^ Yet, insecure attachment styles (especially disorganized) predict maladjustment and psychopathology across the lifespan.^12^, ^34^, ^36^ Fortunately, an individual's attachment style is not absolute, and recent advances in psychotherapy suggest that interventions can help people overcome attachment‐related pathologies.^37^, ^38^

Attachment relationships have many putative benefits for mental and physical health, and having high‐quality relationships predicts longevity.^13^ This association may be, in part, due to the effects of phenomena like social buffering and loneliness (Figure 2). Social buffering is when the presence of social support, (e.g., an attachment partner) alleviates the psychological perception of and neuroendocrine responses to stress and anxiety.^39^, ^40^, ^41^, ^42^ Specifically, there is reduced activation of the hypothalamic–pituitary–adrenocortical (HPA) system (e.g., cortisol release) in response to stressors.^43^ This particular effect is beneficial because long‐term activation of the HPA system can negatively impact cognitive performance and instigate a host of physiological diseases (e.g., heart disease).^44^, ^45^ However, not all attachments provide the same social buffering benefits, and a large contribution of human‐focused research is in showing that *perceived* social support or “connectedness” is key for social buffering and wellbeing generally.^42^, ^46^ Individuals with secure attachments typically perceive more social support and are more satisfied than those with insecure attachments.^47^, ^48^ Moreover, individuals with insecure attachments are more likely to experience social isolation, or loneliness.^49^

Loneliness is itself a major life stressor, and lonely individuals are characterized by negative mental states such as anxiety, depressed mood, low life satisfaction and high sensitivity to social threats.^49^, ^50^, ^51^, ^52^, ^53^ Lonely individuals also have high morbidity and mortality from cancer, cardiovascular disease, high blood pressure and many other health conditions.^50^, ^52^, ^53^, ^54^, ^55^, ^56^, ^57^ Lonely individuals are less likely to actively cope by seeking out support from others, thereby sinking into a vicious cycle of isolation that is difficult to reverse with therapeutic interventions.^50^, ^54^, ^58^

### 3.2 Neural mechanisms of human attachment behavior

Research on the mechanisms of human attachment has focused on identifying associations between attachment styles and broad neural activation in hemispheres, cortical structures and networks.^59^, ^60^, ^61^ Data from electroencephalogram (EEG) studies, for example, including studies of hemispheric asymmetries and event‐related potentials, have shown that these neural patterns vary across individuals as a function of attachment style.^60^, ^62^, ^63^, ^64^, ^65^, ^66^, ^67^ Those data are difficult to relate to results from animal models, however, and are thoroughly reviewed elsewhere.^31^, ^32^ Since the early 2000s, in contrast, fMRI research has expanded on EEG studies by identifying brain networks linked to individual variation in adult attachment.^59^, ^61^, ^68^, ^69^ Such investigations have led to an enriched neuro‐anatomical model of attachment (NAMA) recently articulated by Vrtička and colleagues that not only reviews anatomical signatures of attachment, but postulates a neural framework that lends itself to comparison with data from animal models.^31^, ^32^

Some of the key regions identified include cortico‐limbic networks involved in social reward and motivation like the ventral striatum, ventral tegmental area (VTA), amygdala, anterior cingulate cortex (ACC), medial prefrontal cortex (mPFC) and orbitofrontal cortex (OFC), as well as cortical networks involved in executive function like the superior temporal sulcus (STS), temporo‐parietal junction (TPJ), anterior insula (IA), inferior frontal gyrus (IFG) and supplementary motor area (SMA). Individuals varying in their attachment styles and perception of social isolation show distinct neural signatures, especially when processing social stimuli that has an emotional valence. When processing negative social stimuli, avoidant individuals show suppressed activity in areas of the brain linked to distress like ACC and IA.^70^ On the other hand, anxiously‐attached individuals show amplified activity in the ACC, IA and amygdala,^70^, ^71^, ^72^, ^73^, ^74^ and lonely individuals show amplified activity in the visual cortex.^75^ Also, anxiously‐attached and lonely individuals show suppressed activity in areas of the brain involved in emotion regulation, such as OFC and TPJ.^71^, ^75^ When processing positive social stimuli, lonely individuals and those with avoidant and anxious attachment styles show suppressed activity in brain areas involved in social reward like ventral striatum and VTA.^72^, ^73^, ^75^ Moreover, avoidantly‐attached individuals show elevated activity in areas associated with emotion regulation like OFC.^74^

Another way to investigate cortical underpinnings of social attachment is to assess individual variation in functional connectivity. These types of fMRI studies show that lonely individuals exhibit high connectivity in cingulo‐opercular networks associated with tonic alertness and low connectivity in fronto‐parietal networks associated with executive control.^76^ Conversely, individuals with many social attachments exhibit high connectivity in affiliative (e.g., medial amygdala and ventromedial PFC, striatum and hypothalamus) and perceptual networks (e.g., ventrolateral amygdala, STS and OFC), but not avoidant networks (e.g., dorsal amygdala, insula and ventrolateral striatum).^77^ In sum, these correlational data support the putative hypothesis that distinct top‐down and bottom‐up processes underlie individual variation in the cognitive processing and emotional regulation of social stimuli.^52^, ^61^

Although the majority of research on the biological basis of human attachment centers around activation patterns within specific brain networks, a complementary line of work highlights the importance of neurogenetic mechanisms and biochemical systems.^31^, ^32^, ^59^, ^78^, ^79^, ^80^ Variation in attachment style and related behaviors often reflect variation in genes that regulate neuropeptide receptors, including oxytocin receptor (OXTR) and the arginine‐vasopressin 1a receptor subtype (AVPR1a).^81^, ^82^, ^83^ Specific SNP polymorphisms in the OXTR gene have been shown to predict childhood social problems and adult patterns of pair‐bonding and separation anxiety.^84^, ^85^, ^86^ Similarly, polymorphisms in the APVR1a gene in men predict variation in partner bonding and marital quality.^87^ Neuropeptide system functioning also maps onto variation in social attachment behaviors. Mothers and fathers who are more affectionate towards their infants show elevated plasma oxytocin levels following infant contact.^88^ Adults with higher plasma vasopressin levels report fewer negative marital interactions and greater attachment security.^89^ In sum, research on the biological basis of human attachment behavior has offered important insights into how the functioning of neurobiological systems predict attachment styles and related attachment behaviors. It is difficult, however, to tease apart correlation from causation with human research. For this reason, we can gain important insights from nonhuman animal work, where researchers can thoroughly dissect evolutionary and mechanistic processes.

Recently Vrtička and colleagues have argued for two distinct networks related to human attachment: an affective network that includes brain regions related to positive and negative affect (termed “approach” and “avoidance” modules); and a cognitive network made up of modules dedicated to emotional self‐regulation and mental‐state representation.^31^, ^32^ Their approach module corresponds to a network of brain regions that are generally implicated in positive affect and reward, specifically the ventral striatum/nucleus accumbens, hypothalamus, ventromedial PFC, OFC and VTA/substantia nigra. The aversion module comprises the hippocampus and hypothalamus, the amygdala, anterior temporal pole of the cortex, the insula and the ACC. Unlike the affective network, which is largely but not exclusively subcortical, their cognitive network is entirely neocortical. It includes an emotional self‐regulation module made up of the lateral orbitofrontal and dorsolateral prefrontal cortices, while the putative mentalization module consists of the mPFC, the superior temporal gyrus, STS, the TPJ, the fusiform gyrus and the posterior cingulate cortex. The authors regard secure attachment as prototypical (after earlier work^90^), with derivations based on experience leading to insecure attachments that are either anxious, avoidant, or both (disorganized). A key insight of this taxonomy of attachment and its neural mechanisms are that (1) insecure attachments vary in multiple dimensions that should not be conflated, and (2) that anxious and avoidant attachment can be regarded as adaptive responses to developmental and social settings.^31^, ^32^

The distinction between cognitive and affective networks is a useful synthesis of diverse dimensions of work in human social neuroscience, and the affective networks have logical counterparts in animal research. Animal research, however, enables finer anatomical parcellation than is currently possible in imaging studies, and enables precise causal manipulations. The two thus provide complementary approaches to the understanding of attachment and related behaviors.

## 4 VARIATION IN SOCIAL ATTACHMENT BEHAVIOR AMONG NONHUMAN ANIMALS

The study of bonding and attachment in animals follows in the footsteps of Lorenz and Harlow in the academic disciplines of ethology and comparative psychology (Figure 2). In this approach, research has focused on the evolutionary functions of social bonds and the physiological mechanisms evolved to promote attachment. Below, we first review evolutionary frameworks used to explain why individuals invest in social bonding. Then, we summarize neuroendocrinology research that has advanced understanding of how attachment formation varies across individuals and species. We note also that our use of “attachment” in this context is broader than its use by social psychologists. Where social psychologists and neuroscientists working in that tradition may focus specifically on the mechanisms and uses of proximity maintenance in response to threat, as formulated by Bowlby and Ainsworth,^21^ in work with animals, researchers often refer to attachment more generally as the selective maintenance of proximity between individuals. Such “attachments” are reflected in the partner‐preference tests used to assess pair‐bonding in prairie voles for example.^91^ While the novelty of this test may be sufficient to serve as a threat and elicit proximity‐seeking behaviors, the role of threat in such tests has not been a focus of animal research. We suggest that the active maintenance of proximity assists in the collaborative behavior that defines a bond—a pattern of cooperation that includes but is not limited to responses to threat. Thus attachment and “affiliation” are often treated as synonyms in animal research.

### 4.1 Evolutionary functions of attachment behavior

Social attachments can arise between many types of dyads, including parent–offspring, kin and nonkin. Behavioral ecological explanations of social behavior generally begin with an assumption that individual behaviors and variations in those behaviors attempt to maximize individual fitness.^92^ Parental‐investment theory, for example, proposes that direct parental care (e.g., feeding and carrying) is important for increasing an offspring's chance of survival, which in turn, benefits the reproductive success of the parent.^93^ It is much more common to observe direct maternal care than to observe direct paternal care, presumably because females have greater certainty of their parentage and invest more in fertilization and gestation than do males.^93^ However, there are also many species for which fathers invest in direct care, including several genera of Platyrrhine primates (e.g., *Aotus*, *Callithrix*, and *Saguinus*),^94^ many bird species (e.g., *Actitis*, *Charadrius*),^95^ and fish (e.g., *Syngnathidae* and *Gasterosteidae*).^96^, ^97^ Social attachments between kin also occur outside of the parent–offspring dynamic—such as sibling alloparental care—which can be explained by kin‐selection theory.^98^, ^99^ In other words, attachments may promote investment in kin by contributing to inclusive fitness.

An evolutionary framework also helps explain the function and variation of social attachment behavior between nonkin (Figure 2). One way nonkin social attachment behaviors are expressed is through socially monogamous mating patterns, in which females and males form “pair bonds” and live together for one or more breeding seasons. Social monogamy is rare across the animal kingdom with the exception of avian taxa, and genetic monogamy (i.e., sexual exclusivity to the mating partner) is rarer still.^100^, ^101^ For the taxa that form pair bonds, individuals may gain crucial fitness benefits by cooperating to defend resources and to raise young.

Nonkin social bonds are frequently expressed outside the mating context. It is a challenge to explain the evolutionary function of such social bonds given that the connection to reproductive fitness is not as straightforward. Theories to help explain why the expression of social attachment‐like relationships vary within a group include reciprocal altruism^102^ and biological market theory.^103^, ^104^, ^105^ Reciprocal altruism posits that mutual investment in a long‐lasting relationship is beneficial to both parties because temporary reductions in fitness to engage in affiliative or cooperative behaviors (e.g., alloparenting, food‐sharing and third party conflict support) are gained back when these favors are returned.^106^, ^107^ Similarly, biological market theory posits that cooperative interactions can be explained by game theoretic models in which one behavioral “commodity” is traded for another (e.g., grooming reciprocity in primates^108^). In both cases, these trades are more reliable when individuals cooperate with others with whom they have developed long‐lasting and stable attachment‐like relationships.^103^

One feature all of these kinds of relationships share in common is that they involve the collaboration of two individuals through direct interaction, an interaction that requires some significant degree of proximity.

### 4.2 Mechanisms of attachment behavior in nonhuman animals

Research on the mechanisms of social attachment behavior in nonhuman animals has been largely focused on peripheral and subcortical systems that regulate maternal behavior and pair‐bonding.^109^ The most peripheral of these systems, the endocrine system, includes steroid and protein hormones. Ovarian hormones from the hypothalamic–pituitary–gonadal (HPG) axis—estrogen (e.g., estradiol), progesterone, and prolactin—are thought to prime maternal responsiveness in several species, including rodents, sheep and primates.^110^, ^111^ Secretion of glucocorticoids (e.g., cortisol) from the hypothalamic–pituitary–adrenal (HPA) axis can trigger affiliative behavior and pair‐bond formation in socially monogamous species like common marmosets and prairie voles.^81^, ^91^ Conversely, interactions among individuals can significantly reduce the response to an external stressor—a phenomenon known as social buffering that occurs, not only in humans, but also in a variety of other taxa including prairie voles and nonhuman primates.^40^, ^41^, ^112^

Decades of nonhuman animal research shows that neuropeptide systems, notably oxytocin (OT) and vasopressin (AVP), are involved in individual variation in social behavior.^83^, ^110^, ^111^, ^113^, ^114^, ^115^, ^116^, ^117^ OT administration is shown to stimulate maternal responsiveness in rodents, sheep and primates,^118^, ^119^, ^120^ and circulating peripheral levels of OT are positively correlated with alloparenting behavior in common marmosets.^121^ Moreover, both OT and AVP systems are thought to facilitate affiliative behaviors critical for pair‐bond development in socially monogamous taxa, including new world primates,^122^, ^123^, ^124^ prairie voles^125^ and California mice,^126^ songbirds,^127^ and cichlid fish.^128^

Several subcortical brain structures have been implicated in the regulation of social attachment behavior, many of which contain receptors for the neuromodulator systems summarized above. The medial preoptic area (MPOA) and bed nucleus of the stria terminalis (BNST) are thought to act as central hubs in the “maternal neural network”,^110^ as well as the “social behavior” and “social decision‐making” networks.^129^, ^130^, ^131^ MPOA and BNST express receptors for estradiol, prolactin and OT which, when activated, are thought to initiate maternal behavior via downstream areas like the ventral tegmental area (VTA), nucleus accumbens (NAcc) and ventral pallidum (VP). In rats, for example, MPOA estradiol receptor expression and sensitivity levels correspond to individual variation in maternal styles, with highly attentive mothers having enhanced levels of receptor expression and sensitivity.^132^ In addition, oxytocin receptors in the VTA are thought to play a key role in both maternal care and pair‐bond formation, specifically by interacting with the NAcc dopamine in the mesolimbic “reward” system.^133^, ^134^, ^135^ In rats, pharmacological inhibition of VTA OT receptors decreases NAcc dopamine release in rats, which in turn reduces maternal behavior.^135^ In female prairie voles, pair‐bonding behavior requires activation of both OT and dopamine D2‐type receptors, and pharmacological inhibition of one receptor type prevents the other from having an effect.^133^ In male prairie voles, however, pair‐bonding behavior is enhanced by pharmacological activation of NAcc D2‐type receptors and AVP 1a receptors in VP.^136^, ^137^

The functioning of neuromodulator systems within the brain, and their impact on social attachment behaviors, are sensitive to developmental processes involving genetic and epigenetic programming and environmental influences.^138^, ^139^, ^140^ Polymorphisms in the genetic loci for OT and AVP receptors (i.e., OXTR and AVPR1a) predict intra‐ and inter‐species differences in rodent and primate social behavior.^141^, ^142^, ^143^ Early life experiences (e.g., maternal care) can modify the epigenetic programming of these genetic loci, leading to inter‐generational changes in attachment behavior.^144^ In prairie voles, for example, lower levels of parental care resulted in offspring having enhanced DNA methylation of CpG regulatory sites in the OXTR gene.^145^ With recent advances in tools to manipulate gene expression and edit genomes, it is becoming more feasible to assess the function of OT and AVP receptor genes in regards to attachment behavior.^146^ In prairie voles, for example, knock‐down of NAcc OXTR mRNA in NAcc results in a reduction of alloparental and disrupted partner preference formation.^147^

In sum, nonhuman animal research has offered a unique perspective on evolved mechanisms for social attachment behavior and its variations. Much of this work emphasizes the contribution of biochemical systems (e.g., oxytocin and vasopressin) and their modulation of subcortical neural circuits.

## 5 PRAIRIE VOLES AS A MODEL SPECIES FOR INDIVIDUAL DIFFERENCES

Voles are small microtine (Microtus spp.) rodents found in the grasslands of North America, Europe and northern Asia. Interest in the social behavior of voles, and specifically prairie voles (*M. ochrogaster*), began in the 1970s and 80s when parallel findings from laboratory and field‐based studies uncovered wide variability in vole mating systems.^148^, ^149^, ^150^ Long‐term demographic data showed that prairie voles were more likely to be repeatedly caught in the same male–female pairs than were their sister taxa.^148^ Moreover, paired prairie voles in captivity expressed tolerance towards their mates but were aggressive towards novel opposite sex individuals. This body of work led to the consensus that prairie voles are one of the few mammals that form long‐lasting female–male attachments, or “pair bonds.” Since this discovery, several labs have sought to understand the evolution and neural mechanisms of prairie vole pair bonds, and specifically, of individual variation in bonding behavior.^151^ Here, we review how research on prairie vole pair‐bonding strategies has integrated evolutionary, developmental, and mechanistic approaches to explore individual variation in behavior related to the expression of male–female social attachments.

### 5.1 Individual variation of mating strategies

The formation and expression of pair‐bonding is highly diverse in wild prairie vole populations. Most individuals form pair bonds, in which both partners engage in biparental care and are “residents” in a territory that they defend from intruders. This is not the only strategy available to voles. Up to 45% of males adopt a “wandering” strategy in which they are non‐territorial and unpaired.^150^, ^152^, ^153^ Being unpaired does not mean that an individual will have no offspring. In fact, wanderers sire about a fourth of the offspring in a population.^154^ This means that even though paired individuals experience greater reproductive success, adopting the wandering strategy is a useful alternative. There are also alternative strategies within paired individuals. Up to 25% of paired individuals engage in extra‐pair fertilizations (EPFs), and the number of embryos produced by promiscuous animals is similar to the number of embryos produced by faithful animals.^155^ These observations suggest that alternative mating strategies within paired individuals have similar fitness. These observations also demonstrate that *social* monogamy as opposed to *genetic* (i.e., sexually exclusivity) monogamy characterizes the prairie vole mating system at the species level,^155^, ^156^ while at the individual level, animals fall at different points along this social‐genetic monogamy spectrum.^155^, ^157^, ^158^

An important behavioral component of male alternative mating tactics is how individuals range within their environment. In general, males with larger home ranges have more EPFs.^154^ Resident males with the smallest home ranges tend to sire young exclusively with their partners. On the other hand, resident males with larger home ranges gain EPFs with neighboring females, and in turn, they are more likely to be cuckolded by their female partners. Thus, male space use predicts the mating strategies taken by residents, with consequences for extra‐pair and intra‐pair paternity. Similarly, non‐resident wanderer males with the largest home ranges are the ones that gain EPFs, suggesting that the wandering strategy is successful when males' home ranges overlap with several female home ranges. This high level of diversity in prairie vole pair‐bonding strategies prompts two fundamental questions. What neurodevelopmental mechanisms underpin these alternative strategies, and second, how does natural selection support variation in these neural mechanisms? Below, we review research that addresses these two questions, with a specific focus on the vasopressin system and male social behavior.

### 5.2 Cortical vasopressin and mating strategy

Vasopressin (AVP) is a neuropeptide involved in a wide spectrum of social behaviors, especially among male mammals.^129^, ^130^, ^131^ In voles, species and individual differences in mating strategies reflects subcortical and cortical variation in AVP receptor distribution. As compared with promiscuous species, prairie voles have high AVPR1a expression in ventral pallidum, a region of reward circuitry that supports pair‐bond formation.^159^, ^160^ Antagonizing these pallidal receptors in prairie voles disrupts pair‐bonding in males,^137^ while overexpressing these receptors in promiscuous male meadow voles leads to attachment‐like behaviors.^161^ Although pallidal AVPR1a expression reflects species differences in mating strategy, this variation does not generalize to the individual level. Resident and wanderer prairie voles, as well as paired and unpaired voles, all have similar levels of AVPR1a pallidal expression.^154^, ^162^ This lack of variation is interpreted as having been driven by natural selection on the capacity to form bonds: all prairie vole males have high pallidal V1aR, and all are capable of pair‐bonding. The lack of variation in the pallidum, however, has spurred a focus on the remarkable individual differences evident elsewhere in the brain, and specifically, cortical areas associated with spatial memory.^162^

Retrosplenial cortex (RSC) is part of a larger network that is involved in a variety human cognitive processes, ranging from navigation and episodic memory to future planning.^163^ A growing body of work on prairie voles suggests that AVP receptor expression in the RSC (i.e., posterior cingulate) mirrors individual differences in social motivation, memory and cognition. Male wanderers and resident males with overlapping territories (and more EPFs) have low AVPR1a expression levels in RSC, whereas resident males with little territory overlap (and more IPFs) have greater RSC‐AVPR1a expression.^154^, ^164^ These links between mating strategy and cortical AVP receptor abundance is specific to males.^165^ Taken together, these findings indicate that the AVP system shapes individual differences in socio‐spatial memory and mating strategy. One potential explanation is that males with high RSC‐AVPR1a are better equipped to remember the spatial location of social interactions, and in turn, guard their mates.^158^

### 5.3 Neurodevelopmental processes and cortical AVPR1a

Understanding the origins of behavioral diversity requires research that spans across multiple biological levels. AVPR1a abundance in RSC may exert a direct influence on prairie vole space‐use and sexual fidelity, but how does this cortical variation emerge in the first place? Such cortical variation may originate from early life experiences and biological processes that are genetic, epigenetic, or both.^158^ A large body of rodent work indicates that perinatal interventions can alter neuroendocrine profiles and social behavior as adults.^79^, ^140^ In prairie voles, early life interventions include variation in parental care, social enrichment and external stressors,^166^, ^167^, ^168^, ^169^ as well as biochemical interventions like exposures to exogenous neuropeptides and valproic acid.^167^, ^170^, ^171^ Specifically, biparental care may impact AVP receptor expression in the male prairie vole cortex. One study showed that males raised with a father have lower AVPR1a receptor densities in the RSC than males raised without a father, a endophenotype that mirrors the “wandering” male mating strategy.^172^ One explanation for how early social experiences may translate into later‐life neuroendocrine profiles is that individual genotypes interact with epigenetic programming at the AVPR1a gene locus.^169^, ^173^

Early prairie vole research indicated that differences in the AVPR1a gene (e.g., promotor length) is a putative driver of differences in receptor expression.^174^ A series of subsequent studies show that variation in RSC‐AVPR1a abundance stems from variation in single nucleotide polymorphisms (SNPs) within the AVPR1a gene.^164^, ^173^, ^175^ In brief, four tightly‐linked SNPs are found within *cis*‐regulatory regions of the AVPR1a gene. These SNPs influence epigenetic properties of the locus, for example, by shaping the abundance of CpG sites in a putative enhancer sequence. These genotype differences lead to differences in DNA methylation, and in turn, variation in AVPR1a gene transcription. Two allele types—LO and HI—have been identified. The LO allele has more CpG sites in the putative intron enhancer sequence and is associated with lower RSC‐AVPR1a abundance, as compared with the HI allele. In sum, behavioral variation in mating strategy is partially driven by variation in the abundance of AVPR1a in RSC, and this cortical variation is partially driven by individual differences in genotype and DNA methylation at the AVPR1a locus.

### 5.4 Selection of genetic variation at the avpr1a locus

Given that natural selection acts on heritable traits, the next logical question to ask is whether selection actively maintains variation in genotype. Two lines of evidence suggest that individual variation in RSC‐AVPR1a and mating strategy is supported by selection processes. First, HI and LO alleles are found to have similar levels of reproductive fitness in the field.^164^ Specifically, HI alleles are more fit in IPF contexts in which resident males exhibit high sexual fidelity. Conversely, LO alleles are more fit in EPF contexts, where wanderer or resident males mate with non‐partner females. These patterns suggest that alternative male mating strategies have similar levels of evolutionary fitness.

The second line of evidence comes from data on the frequency of SNPs in the AVPR1a locus.^164^, ^176^ This locus has higher levels of polymorphism—specifically, SNPs that are more likely to reach intermediate frequencies in the population—than is observed in the genome as a whole.^176^ This pattern of molecular variation is a hallmark of natural selection that actively maintains alternative versions of alleles, a phenomenon known as balancing selection. Moreover, this signature of balancing selection is concentrated in gene regulatory sequences known as enhancers that seem to be active in the prairie vole RSC. Together, these lines of evidence suggest that natural selection actively maintains diversity in cortical V1aR abundance in the form of alternative male strategies.^158^

## 6 APPLICATION OF MULTI‐DIMENSIONAL FRAMEWORKS TO ATTACHMENT

As discussed earlier in the review, there are a variety of frameworks for understanding behavior that focus on the notion that “causality” can occur at many levels (Figure 1). Understanding behavior thus requires some specificity about the level of causality investigated, and some humility about the usefulness of other perspectives. There is, however, also value in examining these diverse levels in parallel. Because causal explanations at the level of psychological constructs, for example, must ultimately rest on underlying mechanisms, the two sorts of explanations are not at odds, but rather are logical complements to one another. Indeed, science is always limited by the simplifying assumptions and technical constraints imposed by available tools. Examining behaviors from different levels of causation can test the coherence of our explanations, and this approach also provides a means of checking the shortcomings of one perspective against the very different shortcomings of another. This sort of triangulation to develop robust explanations resembles a contemporary multi‐dimensional approach forwarded by Cacioppo and Decety in social neuroscience, which they have termed the “Golden Triangle”.^177^, ^178^

The goal of the Golden Triangle is to combine a trifecta of analytical approaches to better understand complex psychological constructs relevant in social neuroscience, such as empathy and loneliness. This multi‐tiered approach includes behavioral assessments, physiological measurements, and experimental manipulations (Figure 1). The purpose of behavioral assessments is to develop tasks that break down psychological constructs into smaller components that can be reliably quantified and associated with neural processes. Behavioral assessments include reaction time to stimuli, choice tasks, and questionnaires. Physiological measurements include correlative data operating on a wide range of temporal (millisecond to ontogeny), and spatial (molecules to sociocultural structures) scales. Common correlational measurements include neuroimaging techniques (e.g., EEG and fMRI) and peripheral assays like heart rate variability and neuroendocrine levels. Experimental manipulations are methods used to determine causal effects on various biological, temporal and spatial scales. These methods include TMS, pharmacological treatments, lesions and experiments on nonhuman animal models. This approach is analogous to the endophenotype approach in psychiatry, where intermediate phenotypes for complex neuropsychiatric diseases are described through the synthesis of behavioral symptoms and quantifiable biological traits.^179^

Returning to our perspective on attachment, can we see the effectiveness of such multi‐dimensional approaches? What insights do these approaches provide, and what remains to be done?

The history of work on attachment has revealed the value of multidisciplinary perspectives. As we have discussed, the ecological and evolutionary study of imprinting and parent‐offspring attachments led directly to the intellectual breakthrough that the love between parents and their children is not a mere secondary consequence of food or comfort.^15^, ^20^ More recently, insights from prairie voles enabled detailed mechanistic explorations of the roles of specific brain regions and neuromodulators on the formation of bonds.^109^ This work directly inspired social psychologists and others to look below the cortex, into reward regions, where they found the predicted correlates of natural attachments.^59^ Indeed, evolutionary first principles suggest that the reward system should be essential to any social bond. The reward system keeps score of whether a behavior is likely to be in one's best interest: it is reward that drives animals to be close to one another. The history of research on attachment shows a productive dialog between molecular, neural, psychological and evolutionary levels of analysis.

Perhaps a major new area for synthesis remains in the study of attachment styles. Here, psychologists have defined natural patterns of individual differences, and linked them both to social environments and to neural function.^61^, ^180^ Insecure attachment styles pre‐dispose people to negative social interactions and poor health outcomes, and are often viewed as pathological.^12^, ^40^ And yet, insecure attachment styles are quite common, occurring in roughly one‐third of children.^181^ Perhaps pathology is not the right framework, or at least not the only framework, from which to view these styles.

**FIGURE 3:**
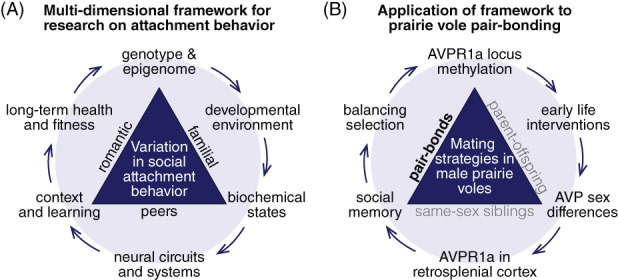
A multi‐dimensional conceptual framework to explain individual variation in social attachment behavior. (A) A schematic of the general framework is shown on the right, which represents different relationship types in the center triangle and biological levels on the outer circle. (B) This framework is adapted to a specific example on male pair‐bonding behavior in prairie voles, the vasopressin (AVP) system and a specific receptor subtype (AVPR1a)

We propose an integrative framework that approaches attachment behavior from multiple biological and social levels, merging traditions from human and animal‐focused research (Figure 3). One strength of the nonhuman research on social attachment is the sheer number of ways that researchers can causally link behavior to mechanisms. These mechanisms include, but are not limited to, genetic programming and developmental processes, biochemical and neural system functioning, contextual plasticity and learning, as well as long‐term fitness consequences and evolutionary outcomes (Figure 3A). Work in animal behavior emphasizes that the fitness benefits of social behaviors often depend on the behaviors of others, and selection can actively maintain alternative social strategies. We have seen that among prairie voles, there are different mating strategies used, and selection actively maintains these differences.^164^ Animals who engage in extra‐pair paternity are not disordered in any meaningful sense. The multi‐scale examination of this causality, that includes field experiments, measures of fitness, measures of gene regulation, and assessments of the long history of selection written in DNA diversity patterns all provide a cogent perspective that individual differences in this dimension are alternative means of success (Figure 3B).

Work on human behavior emphasizes styles of attachment across the lifespan, from early life relationships with caregivers to social bonds between peers and romantic partners (Figure 3A). We suggest that individual differences in attachment styles might be viewed as alternative attachment strategies that are valuable in different contexts. This interpretation parallels the examination of loneliness as an adaptive response to social isolation, one that transitions from connection seeking to a more defensive posture as the likelihood of a useful bond becomes less likely. If this is true, one might expect to find attachment styles in a variety of social species. Indeed, attachment styles have been reported not only in humans, but in rhesus macaques,^182^ domestic dogs,^183^, ^184^ and even cats.^185^ This interpretation accords with many contemporary accounts of human attachment styles, in which anxious or avoidant attachment styles are regarded as functional responses to variation in social experience.^31^, ^32^ It is also consistent with an increasing recognition that temperament may contribute to dimensions of attachment without regard to experience, and that attachment styles may be updated in light of new experiences or the qualities of specific relationships.^186^ The perspective from animal behavior offers another novel and very explicit prediction regarding individual differences in attachment. To the extent that attachment styles have some heritable variation identifiable in suitably powered genome‐wide association studies, the DNA polymorphisms associated with such styles should exhibit signatures of balancing selection, much as does the AVPR1A locus of prairie voles^164^, ^176^ (Figure 3B). Such approaches have been used to identify selection favoring the diversity of human faces, for example, in the signaling of individual identity.^187^ Such analyses would complement other ideas about the maintenance of attachment diversity, such as the social defense hypothesis of Ein‐Dor and colleagues.^188^

Within the more limited perspective of neural mechanisms of attachment, consideration of animal and human data together suggest fruitful areas for future research. In the NAMA model, for example, the affective networks derived from human studies map well onto networks implicated in animal bonding.^31^, ^189^ For example, the “approach module” outlined by the NAMA model includes the ventral striatum, prefrontal cortex, orbitofrontal cortex and the ventral tegmental area/substantia nigra.^31^ In prairie voles and other rodents, the pathways implicated in social reward include most of these structures, as well as others, and offer greater resolution. For example, in the ventral striatum and nucleus accumbens, calcium‐imaging work on prairie voles reveals that this structure contains neurons that are active just before social approach, and that the number of such neurons grows with the formation of a social bond.^190^, ^191^ Similarly, optogenetic manipulations of the coupling between nucleus accumbens and prefrontal cortices shape the formation of prairie vole bonds.^192^ While the affective network of the NAMA model agrees in many respects with perspectives derived from rodent work, in others it seems to diverge. In the NAMA model, for example, the anterior cingulate and insular cortices are assigned to the aversion module, but in prairie voles oxytocin seems to act in these brain regions to promote bonding.^112^, ^193^ In a study of empathy‐like behavior in prairie voles, oxytocin in the anterior cingulate seemed to modulate the ability of consolation grooming to reduce partner stress.^112^ Similarly, rodent work suggests heterogeneity in the functions of hypothalamic and amygdala contributions to positive and negative behaviors that are not entirely consistent with their assignments in the NAMA model (though this likely reflects a limitation of the precision of imaging). Specifically, medial amygdala—preoptic area connections contribute to social reward and affiliative responses, and as such would not belong in the aversion module. Such discrepancies suggest productive areas of research for human neuroscience.

The NAMA model also suggests novel directions for rodent research. For example, common models of pair‐bonding focus on positive affect^189^; incorporation of negative affect would significantly enrich this perspective. In both mice and humans there is a pathway including substantia nigra and ventral tegmental area that regulates negative affect associated with loneliness.^194^, ^195^ This path has been suggested to part of a “social homeostasis circuit”^196^; perhaps it is also involved in the drive to approach specific partners. Another suggestion that derives from studies of human attachment concerns the importance of threat in proximity maintenance. Stress hormones like cortisol are known to promote bond formation in prairie voles,^81^, ^91^ but it is not clear whether acute threats promote proximity‐seeking. Understanding such mechanisms would likely enhance our understanding of attachment in both humans and nonhuman animals.

One last difference between human and animal studies also suggests interesting possibilities for future work. What role, if any, do cognitive processes play in the attachment behaviors of nonhuman species? Are there analogs or homologs of the substrates of mentalizing and self‐regulation that might be at play in the behavior of nonhuman species? For rodents, one is tempted to assume that there are not. However, the demonstration of empathy‐like behavior in prairie voles^112^ and the remarkable degree to which animal work has informed our understanding of affective dimensions of human love suggest that this possibility should not be disregarded a priori. These are, as we have emphasized, distinct research traditions, and where direct comparisons have been possible, there has been a surprising congruity.

## 7 CONCLUSIONS

We have surveyed a broad range of intellectual frameworks—and propose a new framework (Figure 3)—for thinking about the causes of social behavior. These include the examination of multiple levels of analysis as enumerated by scholars dating back to Aristotle. We have described how the distinct intellectual traditions of researchers focused on different levels of causation can offer insights that lead to productive areas of inquiry. Using the case of social attachments, we argue that the history of this discipline is rich with such examples, dating back to its very founding. Contemporary animal studies on the mechanisms and evolution of attachments offer new insights and new avenues of inquiry for the study of human bonds. Social psychology, in contrast, with its detailed examination of human commonalities and differences, also offers new behavioral phenotypes, new intellectual constructs, and new neural substrates for exploration by evolutionary biologists and comparative psychologists. In these senses, the traditions followed by these diverse communities of scientists are poised for intellectual cross‐fertilization and synthesis. Indeed, the study of attachment is a case study in the usefulness of integrative and interdisciplinary approaches.

## Data Availability

Data sharing is not applicable to this article as no new data were created or analyzed.
